# Effects of nanosilica on the properties of brine-base drilling fluid

**DOI:** 10.1038/s41598-023-47932-w

**Published:** 2023-11-22

**Authors:** Peng Xia, Yiheng Pan

**Affiliations:** 1 College of Harbour and Coastal Engineering, Jimei University, Xiamen, 361021 Fujian China; 2 Xiamen Key Laboratory of Green and Smart Coastal Engineering, Xiamen, 361021 Fujian China

**Keywords:** Civil engineering, Nanoscale materials

## Abstract

In the process of drilling oil and gas wells, the shrinkage and falling of wellbore walls are often caused by the expansion of mud shale water. To date, conventional additives have been unable to plug the pore throats of shale rock with nanoscale pores and thus cannot effectively solve the problem of wellbore instability encountered in shale formation drilling. In view of this situation, the idea of using nanosilica to plug the nanopore throat of mud shale is proposed to reduce its permeability and to slow water intrusion. The influences of nanosilica on the properties of brine-base drilling fluid drilling fluid are evaluated by measuring the viscosity, filtration loss and swelling of the drilling fluid. The results of laboratory experiments show that the improvement in drilling fluid properties can only be achieved on the basis of salt resistance; that is, a salt-resistant soil slurry should be used. A concentration of 1–5% nanosilica can improve the viscosity of the drilling fluid by increasing the internal friction between particles. However, nanosilica materials are sensitive to salt concentration. Nanosilica particles can be deposited on the surface of a filter cake to block the pores of the filter paper, and the filtration loss reduction rate can reach 40.2%. Blocking the pores of the clay plays a role in preventing the clay from absorbing water and expanding. The optimal addition amount of silica is 3%, and its salt resistance can reach 16%. Considering the experimental results of filtration loss, swelling amount and cost performance, 3%NP + 4%NaCl + SWM-B is selected as the optimal formula. The results of this study can be applied to effectively improve the phenomenon of wellbore instability during drilling in shale formations, and it has important application value.

## Introduction

In conventional oil and gas drilling, shale and clay formations account for approximately 75% of the total number of wells drilled, and in shale and clay formations, approximately 70% of borehole instability is associated with shale instability^1^. Drilling operations require the right drilling fluid to stabilize shale formations near the borehole, which are prone to expansion and collapse due to interactions with water in the drilling fluid^1^. This problem is common when drilling in shale formations where water-based drilling fluids are used. This increased occurrence is because the water component of the fluid interacts with the shale formation minerals, resulting in the hydration, disintegration, and dispersion of the formation particles^2–4^ this phenomenon causes the clay minerals in the shale to hydrate and swell, leading to wellbore instability, such as wellbore collapse, necking, and pipe jamming^5,6^. One of the most important problems in drilling in shale formations is the instability of shale formations, which poses an enormous challenge to the oil and gas drilling industry^7,8^. Previous studies have focused on improving the stability of shale walls through the use of a variety of treatment agents with strong inhibition and collapsibility^9^. Existing studies have shown that nanoscale and microscale pores or fractures are developed in shale gas reservoirs in China^10,11^, the pore range is 5 ~ 300 nm, the main body is 80 ~ 200 nm^12^, and the pore throat size is 5 ~ 50 nm^13^. To date, conventional additives have not been able to plug nanoscale pore throats of shale, thus forming a thin and dense mud cake^1,7,14^. Drilling fluid intrudes shale formations through these microchannels, further exacerbating shale hydration^5^.

Nanofluids have been used in a wide range of oil and gas wells, from drilling to reservoir, cementing, enhanced oil recovery, completion, and processing^15,16^. Nanoparticles have proven to be more stable than other chemicals during implementation, in addition to significantly improving drilling fluid quality. Nanomaterials have great potential to improve new fluids and devices in the oil and gas industry. Drilling fluid is one of the most challenging areas for nanomaterials to improve their properties. However, the role of nanoparticles in this field is still in its infancy and has therefore attracted considerable attention over the past few years^17^. Nanomaterials such as nano-SiO_2_^18^ and laponite^19^ are beneficial for shale inhibition due to their strong adsorption ability and pore plugging performance, among other advantages^20^. Therefore, nanosized particles are introduced as bridging particles to successfully plug these orifice throats. By comparing nanosilica, nano-TiO_2,_ and nano-Al_2_O_3_ as additives, we find that nanosilica is more effective in reducing liquid loss than nanotitanium and nanoalumina^21^.

Many experiments have been carried out to improve the properties of water-based drilling fluids with nanosilica materials^22^. By comparing the changes in viscosity of water-based drilling fluid under the action of nanosilica particles with different concentrations, it is believed that silica nanoparticles improve the rheological properties of water-based drilling fluid and increase the viscosity and yield point of water-based drilling fluid by more than 50%^23^. verified through laboratory experiments that the addition of nanosilica increases the viscosity of water-based drilling fluid, thus improving the sand carrying capacity and circulation capacity of mud. In addition, nanosilica water-based mud exhibits good mud stability and has a high tendency to prevent formation fluid intrusion. Al-Yasiri et al.^24^ developed and experimentally tested a novel drilling fluid formulation using a mixture of biopolymer nanosilica particles that can effectively improve the performance of water-based mud, enhancing the rheology, filtration and lubrication properties. Liu et al. reported a novel core–shell structure polymer (NS-DA) based on modified silica nanoparticles as a reducing agent for water-based drilling fluids. The experimental analysis shows that the thermal decomposition temperature of NS-DA is 293 °C. Moreover, the material has outstanding filtration reduction ability^25^. Sajjadian et al. researched the improvement in the properties of water-based drilling fluids by three nanomaterials: zirconia, multiwalled carbon nanotubes and titanium dioxide nanohybrids. The rheological properties, filtration properties and conductivity experiments show that the mixed nanomaterials and zirconia can effectively improve the rheological parameters of mud, such as plastic viscosity, yield point and gel strength^26^.

In addition, when drilling projects are carried out in shale formations, conventional drilling fluids cannot effectively maintain the stability of shale. Adding the right number of nanoparticles to water-based mud can not only improve the rheological and thermal properties but also improve borehole stability of the mud^1^. Liu et al. synthesized nanosilica/cationic polymer composites (NS-DD) and evaluated them as novel shale inhibitors in water-based drilling fluids. The nanodioxide composite has significant advantages in terms of high temperature inhibition and cutting recovery^27^. Huang et al.^5^ described hydrophobic nanosilica for strengthening well walls when using water-based drilling fluids. The borehole strengthening performance of this material was studied through a linear expansion test, hot rolling recovery test and compressive strength test^1^. The inhibition effect, inhibition mechanism and advantages and disadvantages of different nanocomposites on shale were introduced. Wei et al. proposed a high-performance water-based drilling fluid suitable for the shales of a well support formation. The rheological property, filtration property, lubrication property, temperature resistance, corrosion inhibition property, pollution resistance and cost of this kind of water-based nanosilica drilling fluid were comprehensively evaluated. Nanosilica drilling fluid has advantages in terms of high temperature resistance, inhibition of shale hydration and operating cost^28^.

To date, there are abundant studies on the effects of nanosilica materials on the properties of water-based drilling fluids and the inhibition of shale, and some achievements have been made. However, there are few studies on the effects of nanosilica materials on the properties of brine-based drilling fluids. Salt pollution has become the most serious challenge for water-based drilling fluids under extreme salinity conditions, which can lead to the deterioration of rheological and fluid loss properties, resulting in drilling accidents, such as leakage, formation collapse and borehole instability^29^. We analyse and evaluate the effects of nanodioxide particles on the properties of brine-based drilling fluid by conducting viscosity testing, filtration loss testing and expansion experiments.

## Experimental materials and methods

### Experimental materials

The materials used in this experiment were saline-resistant soil produced in Anhui Province and bentonite produced in Shandong Province. Xanthan gum (XC) was used as the treating agent. The modified starch (DFD-140) was made in Beijing. The nanosilica material used in the experiment was the nanosilica dispersion liquid produced in Hangzhou. The model was VK-S01B, the average particle size was 15 ± 5/nm, the content of nanosilica was 30%, the solvent was water, and the pH value was 9–11.

### Experimental methods

Two brine drilling fluids, brine-base drilling fluid A (SWM-A) and brine-base drilling fluid B (SWM-B), were formulated according to the slurry configuration method, combined with specifications and references to existing research results. The final test drilling fluid formulations were as follows:

Brine-base drilling fluid A (SWM-A): 350 mL water + 10.5 g sodium bentonite + DFD-140 1.5% + 0.1% XC + 0.32 g NaOH.

Brine-base drilling fluid B (SWM-B): 400 mL water + 16 g anti-salt soil + DFD-140 1.5% + 0.3% XC + 0.32 g NaOH.

The base slurry was prepared according to the selected formula, and different contents of salt were added to the base slurry in six groups of 0%, 4%, 8%, 12%, 20%, and 30%, while four groups of different contents of nanosilica materials 0%, 1%, 3%, and 5% were added. Since the silica dispersion itself contained water, the same amount of water was added to the drilling fluid to eliminate the effect of water, which was represented by extra water. If the slurry pH was small, the pH was adjusted to 9–10 by adding an appropriate amount of sodium carbonate. Wu et al.^30^ stated that when the slurry pH was adjusted to 9–10, the purpose was to allow sufficient hydration and dispersion of the bentonite. A total of 42 sets of experiments were performed. The density, plastic viscosity (PV), dynamic shear (YP), and water loss (API-FL) of the corresponding drilling fluid were tested.

### Stability observation experiment

**Figure 1:**
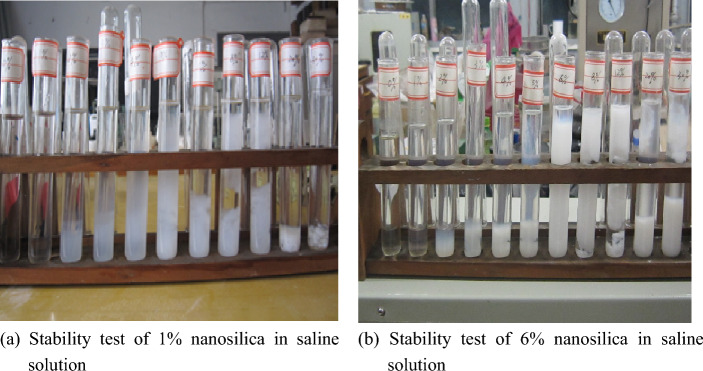
Stability observation test results.

In the test tube, 0%, 1%, 2%, 3%, 4%, 5%, 6%, 8%, 12%, 20%, and 30% saline solutions were prepared, and 1% and 6% nanosilica materials were added to investigate the colour, flocculation, and dispersion layer of the mixing system. The results showed that the salt resistance of nanosilica material was strong when the concentration was low, but the salt resistance decreased when the concentration was too high. The results showed that the salt resistance of the nanosilica material was 6%. Nanosilica materials had small particle sizes and large specific surface areas. Under the condition of high salt concentration, it was found that aggregation, flocculation, and stratification appeared. In addition, with the increase in the concentration of nanoparticles in the mixed system, the thermal motion was intensified, and the particles easily stratified and precipitated. The test results are shown in Fig. 1 below.

## Experimental results and analysis

### Effect of nanosilica concentration on brine-base drilling fluid A (SWM-A) performance impact

The NaCl concentration was adjusted from 0 to 30% by adding 1%, 3% and 5% nanosilica to the saline water-based pulp to explore the improvement effect of nanosilica on the properties of SWM-A.

In the following figures, PV represents plastic viscosity, AV represents apparent viscosity, and FL represents water loss. The amount of water added to the base slurry should be equal to the amount of water contained in the nanosilica dispersion.

**Figure 2:**
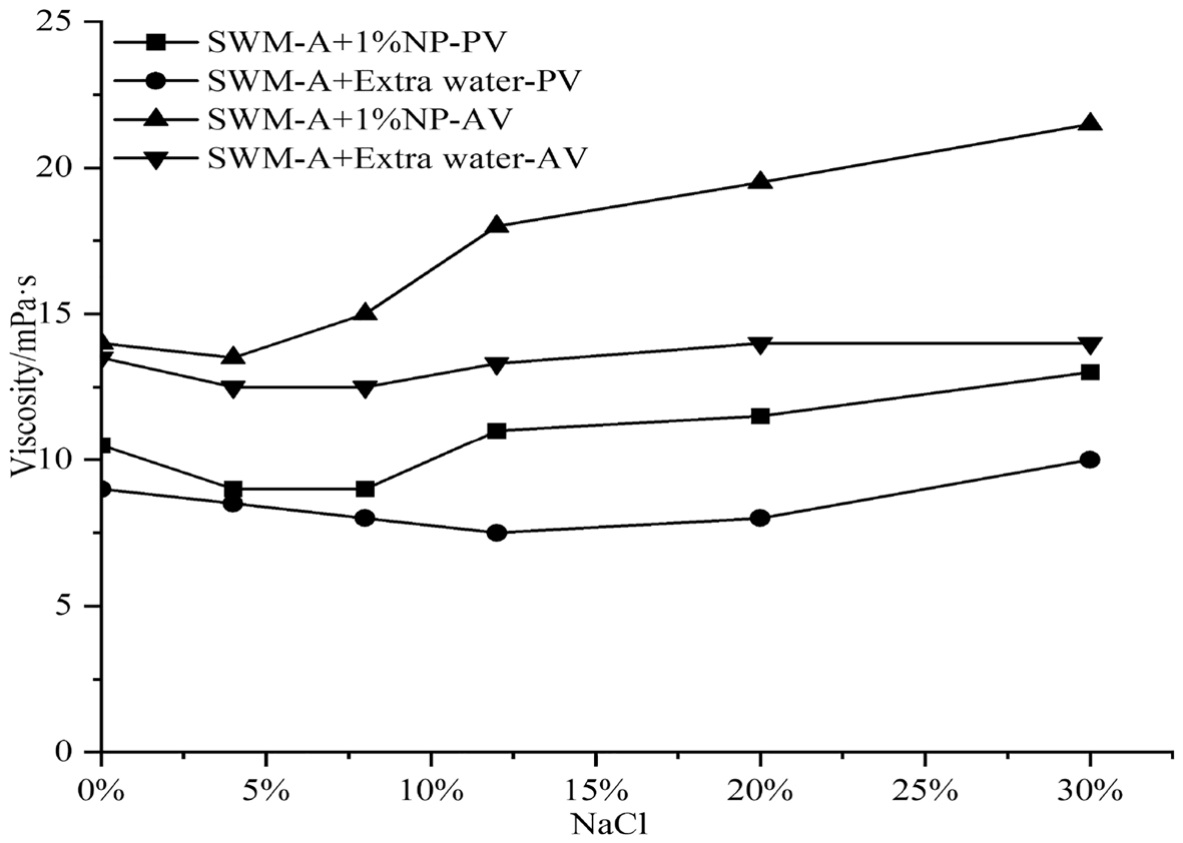
Effect of 1% nanosilica on the viscosity of SWM-A.

**Figure 3:**
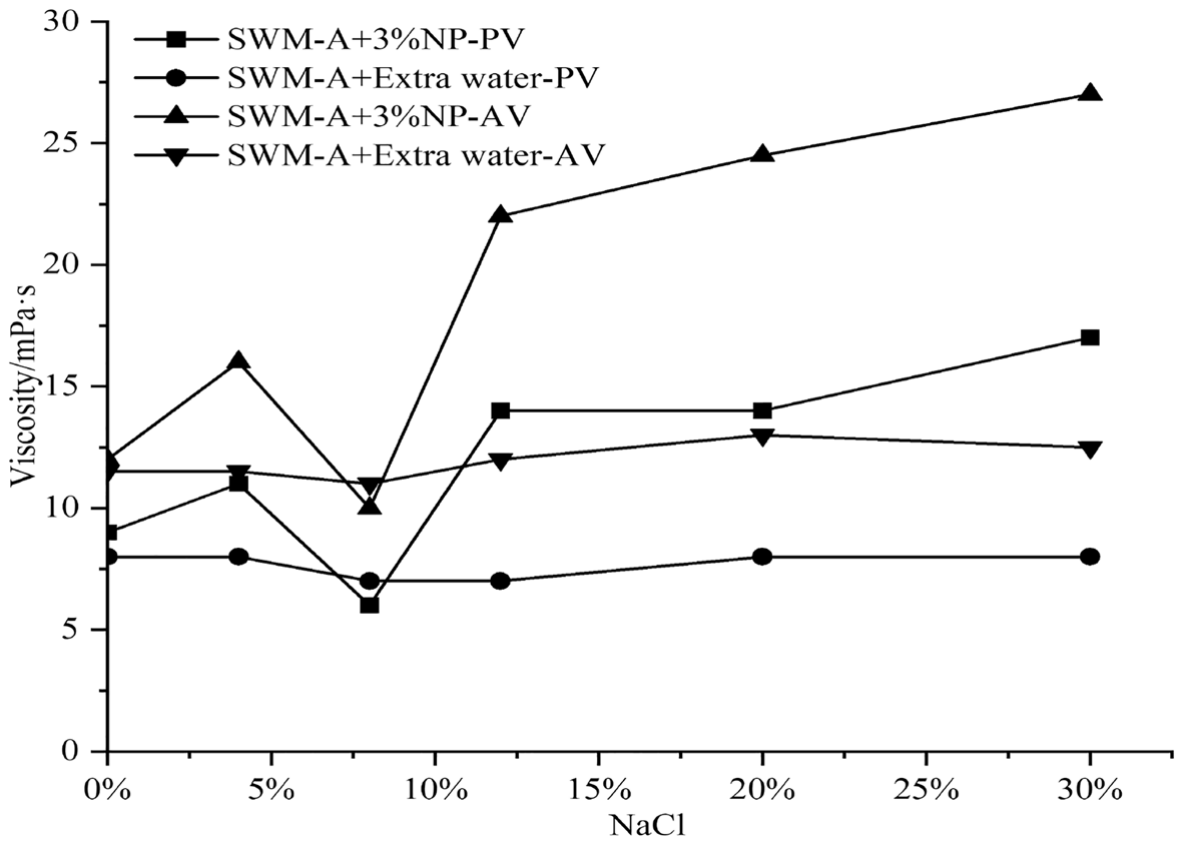
Effect of 3% nanosilica on the viscosity of SWM-A.

**Figure 4:**
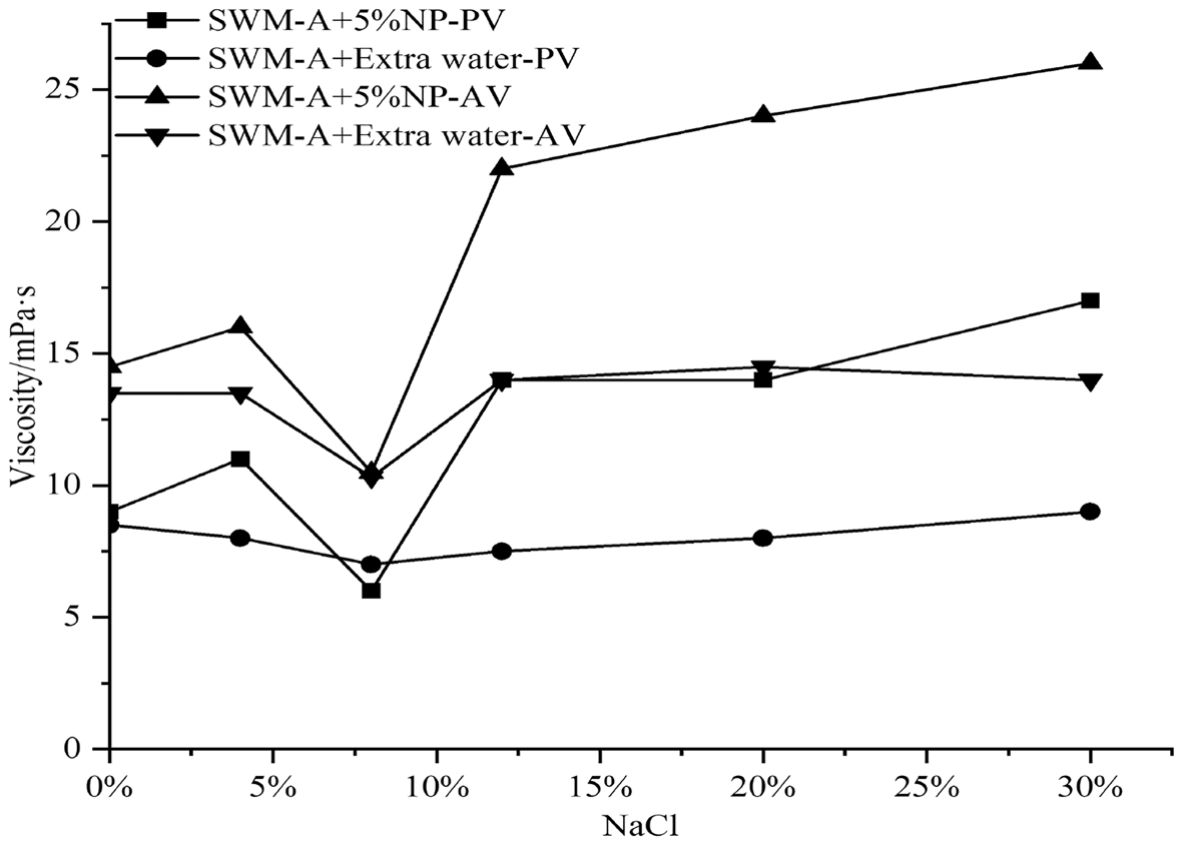
Effect of 5% nanosilica on the viscosity of SWM-A.

**Figure 5:**
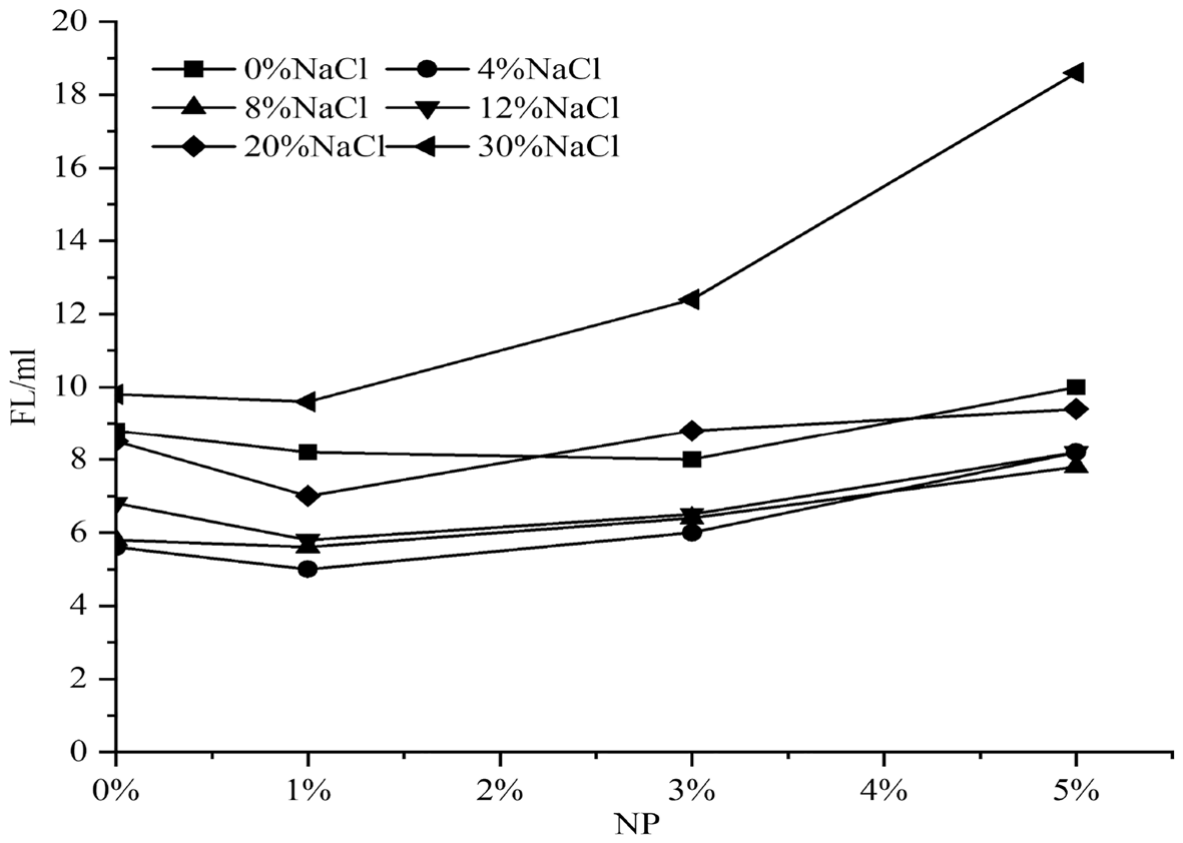
Effect of nanosilica concentration on SWM-A filtration.

Figures 2, 3 and 4 show that with increasing NaCl concentration, PV and AV tend to increase after weakening when the concentration of nanosilica was 3% or 5%. When the NaCl concentration in saltwater drilling fluid exceeded 4%, PV and AV showed sudden decreases, and when the NaCl concentration reached 8%, PV and AV values reached the minimum value. When the NaCl concentration was between 8 and 12%, PV and AV showed rapid increases, and when the NaCl concentration exceeded 12%, they showed slow increases with increasing salt concentration. Figure 5 shows that with increasing nanosilica concentration, the filtration loss of the SWM-A drilling fluid did not decrease but instead increased, and the negative effect was obvious. Although nanosilica particles could increase the viscosity of the SWM-A drilling fluid, it was unstable and had obvious fluctuations. SWM-A drilling fluid had an obvious negative effect on the control of filtrate loss. Therefore, nanosilica materials did not play an effective role in improving the performance of SWM-A drilling fluid.

### Effect of nanosilica concentration on brine-base drilling fluid B (SWM-B) performance impact

To form a comparative experiment and obtain the improvement effect of nanosilica material on drilling fluid, it was necessary to conduct a performance test on the untreated drilling fluid. The density of the untreated drilling fluid was 1.03 g/cm^3^, the plastic viscosity was 11 mPa·s, the dynamic shear force was 6.5 Pa, the API filtration capacity was 17 mL, and the pH value was 11.

#### Effect of salt concentration on the properties of brine-base drilling fluid B (SWM-B)

**Figure 6:**
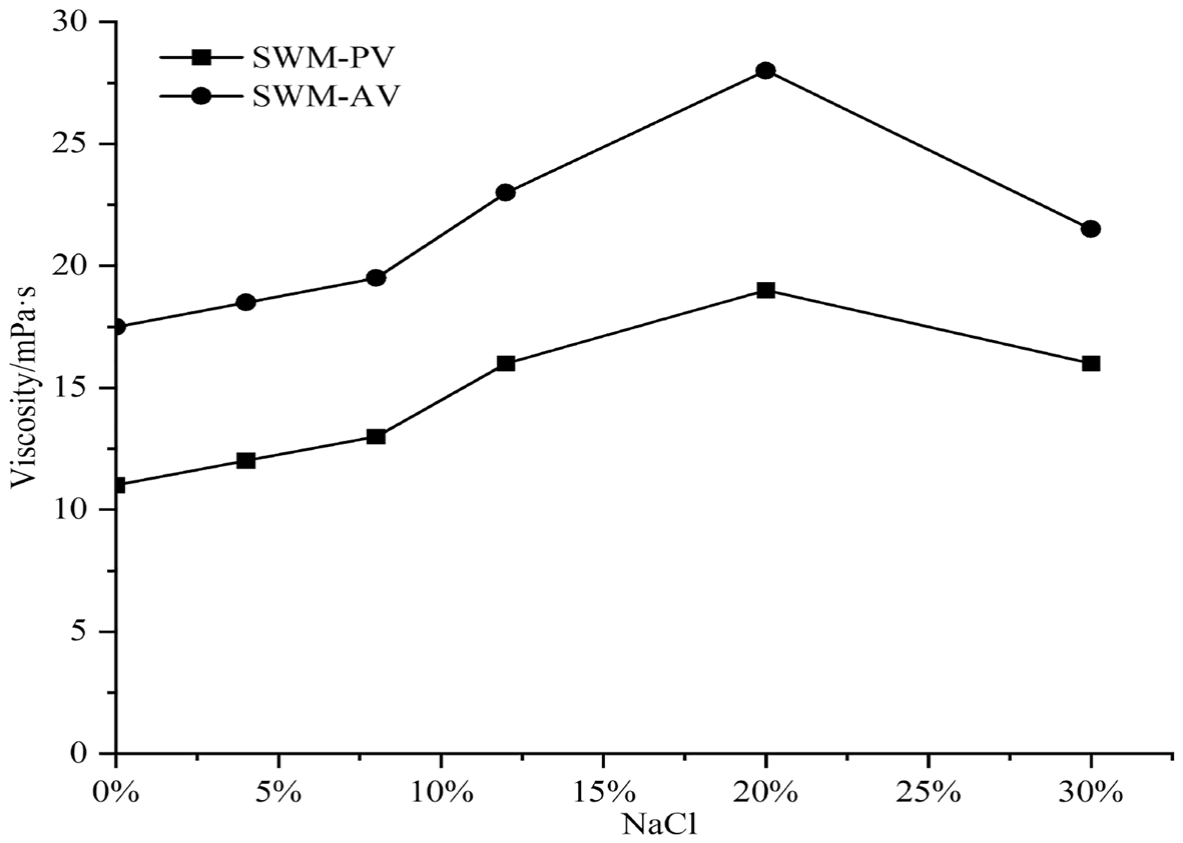
Effect of NaCl concentration on the viscosity of SWM-B.

**Figure 7:**
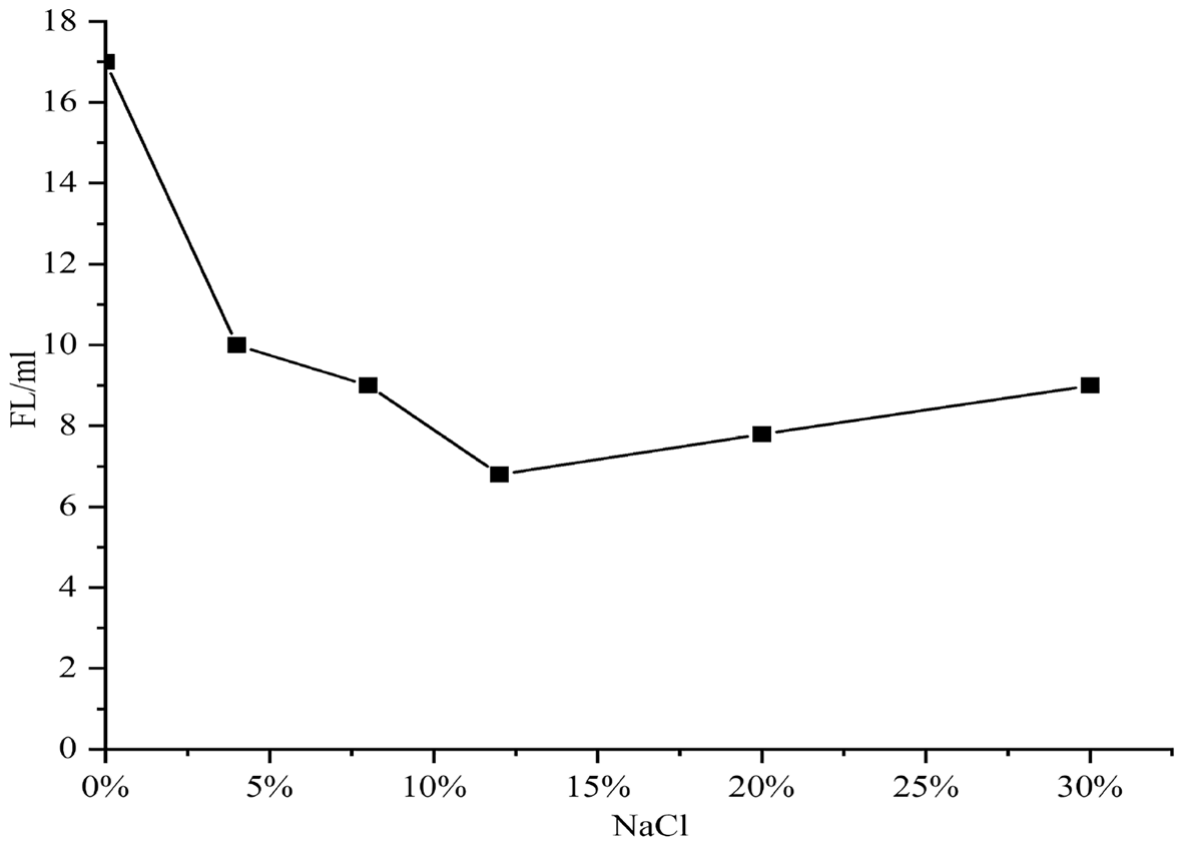
Effect of NaCl concentration on filtration of SWM-B.

By changing the concentration of NaCl in brine drilling fluid B, the measured changes in the filtration and viscosity of SWM-B were determined, as shown in Figs. 6 and 7, respectively.

As seen from Figs. 6 and 7, with increasing NaCl concentration, the viscosity of brine drilling fluid SWM-B first increased and then slowly decreased with increasing NaCl concentration, and its filtration loss showed a feature of first significantly decreasing and then slightly increasing. The reason for this phenomenon was that the degree of hydration of saline-resistant soil in brine was gradually sufficient, its viscosity gradually increased, and the filtration loss significantly decreased. However, as the salt content continued to increase, Na + began to compress the double electric layer, inhibiting the further progression of hydration. As a result, the viscosity of SWM-B began to decrease slowly, and the filtration loss slowly increased.

#### 1% nanosilica effect on SWM-B properties

**Figure 8:**
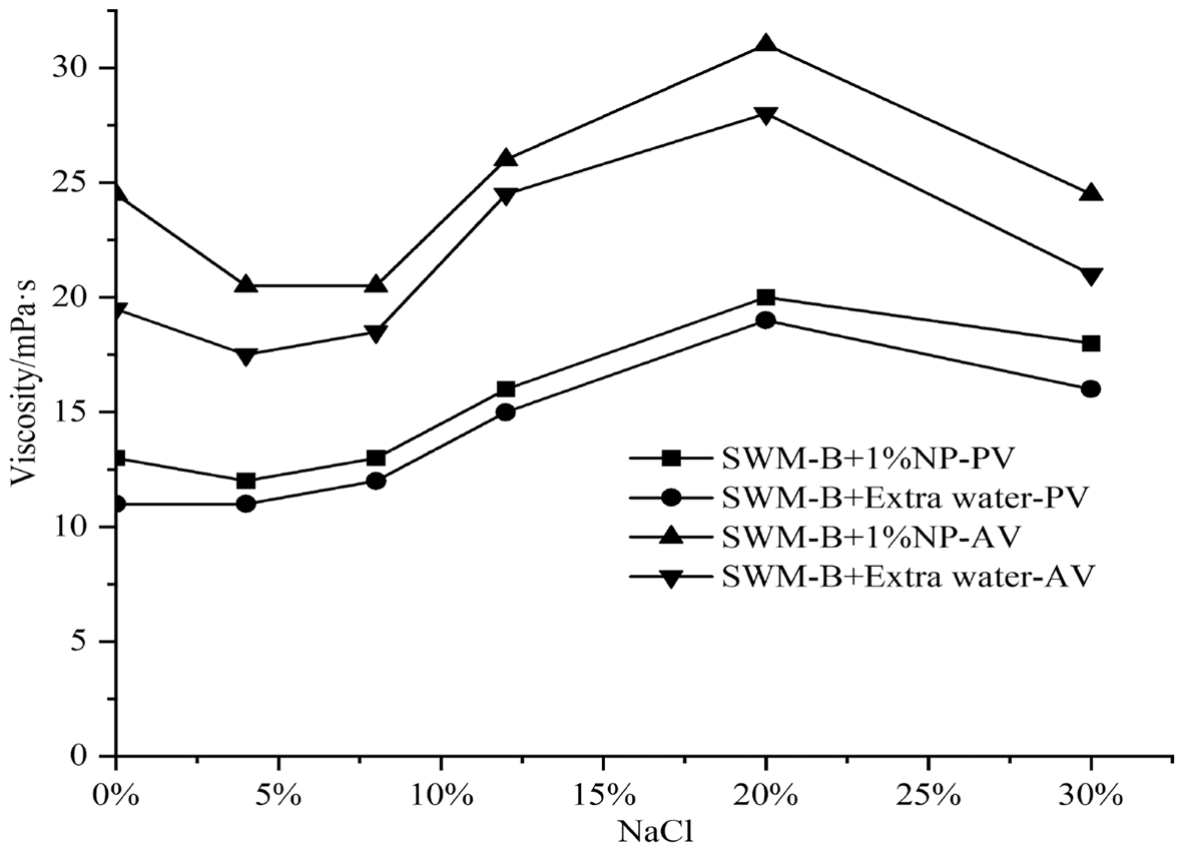
Effect of 1% nanosilica on the viscosity of SWM-B.

**Figure 9:**
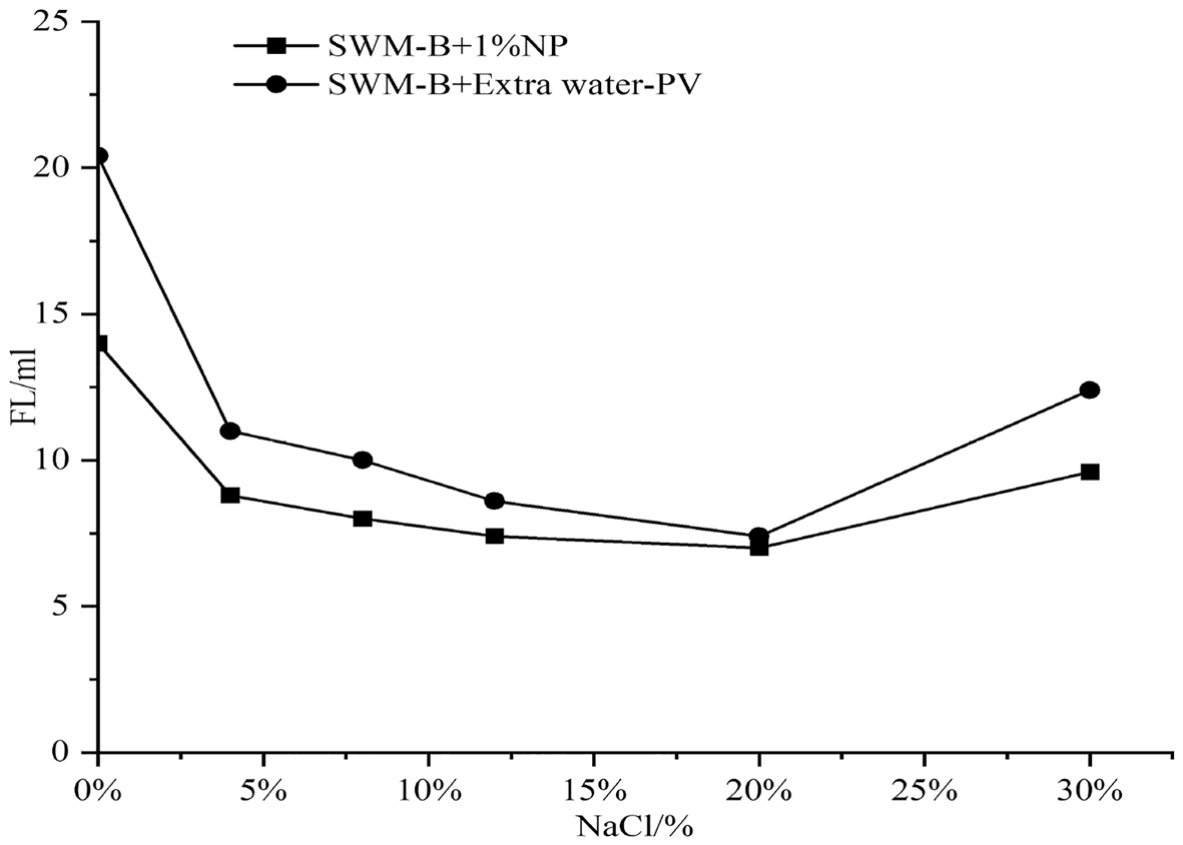
Effect of 1% nanosilica on filtration of SWM-B.

SWM-B was supplemented with 1% nanosilica, and the viscosity and filtration test results were obtained, as shown in Figs. 8 and 9.

As shown in Figs. 8 and 9, when the NaCl concentration was lower than 20%, the viscosity of SWM-B containing a 1% concentration of nanosilica increased with increasing NaCl concentration, and the filtration loss presented a significant decreasing trend. However, when the concentration of NaCl exceeded 20%, the viscosity presented a slow decline with increasing salt concentration, and the filtration loss presented a significant increasing trend. Nanosilica particles could increase the internal friction of the drilling fluid and be deposited on the mud cake surface to seal the filter paper to reduce filtration loss. The performance of SWM-B was significantly improved with a 1% concentration of nanosilica, especially at a low concentration of NaCl.

#### 3% nanosilica effect on SWM-B properties

**Figure 10:**
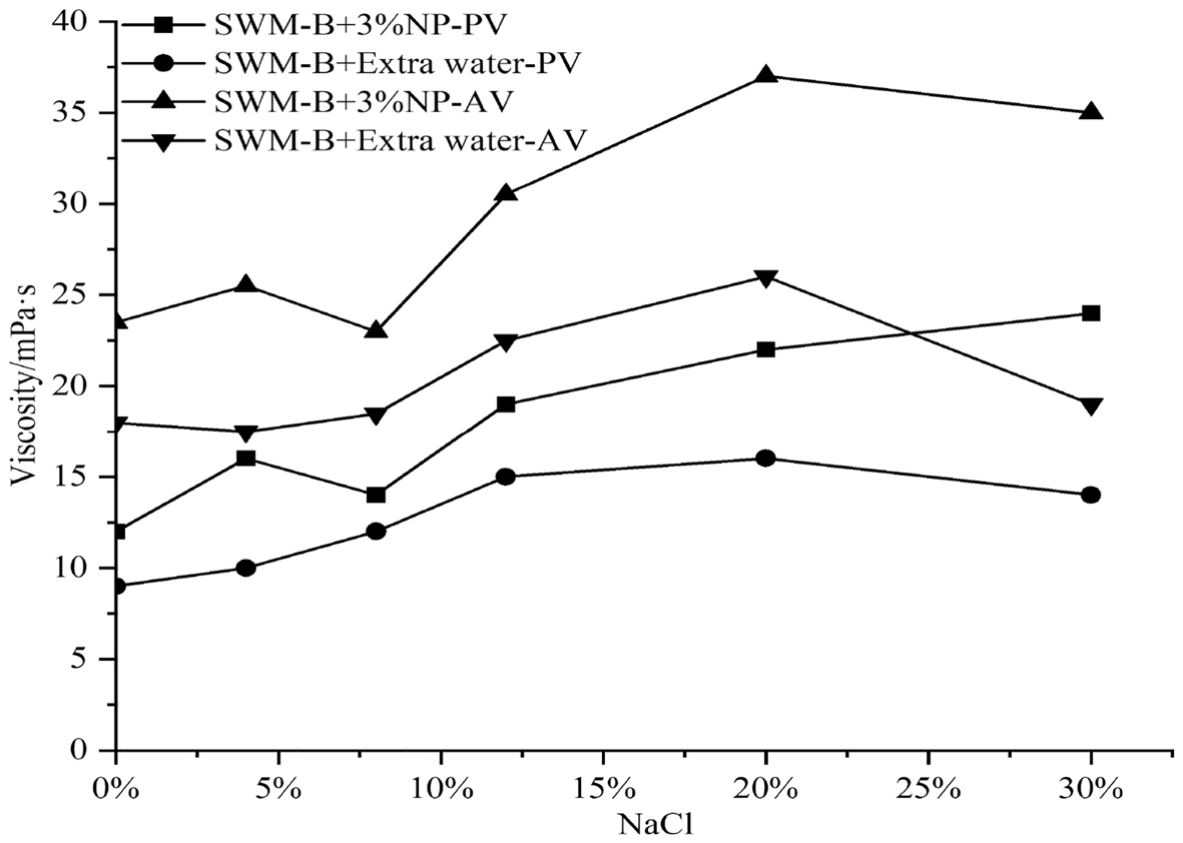
Effect of 3% nanosilica on the viscosity of SWM-B.

**Figure 11:**
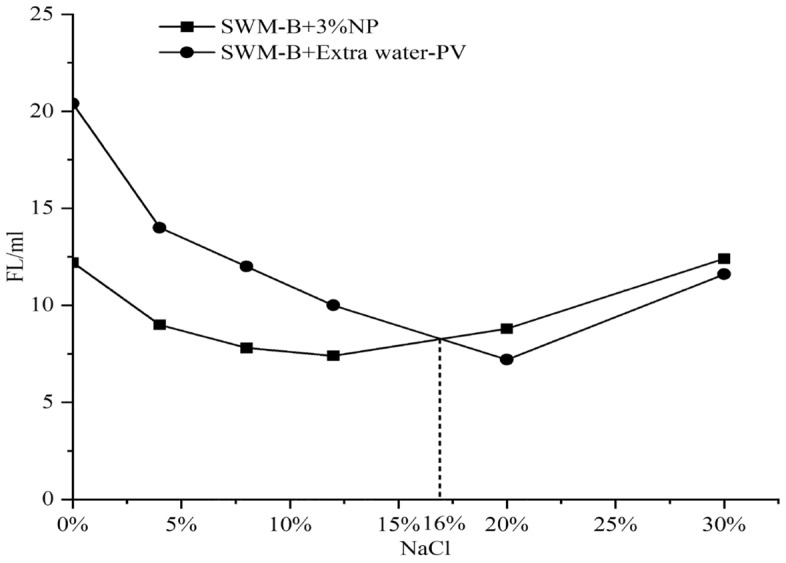
Effect of 3% nanosilica on filtration of SWM-B.

The results of the viscosity and filtration loss experiments are shown in Figs. 10 and 11. Figures 10 and 11 show that the effect of nanosilica on the viscosity of SWM-B was similar.

When the concentration of NaCl was lower than 20%, the viscosity of SWM-B showed an obvious increase with increasing salt concentration. With the salt concentration exceeding 20%, the increase rate of viscosity decreased and stabilized. Under the condition of a low concentration of NaCl, 3% nanosilica had a significant filtration loss reduction ability, and the maximum filtration loss reduction could reach 40.2% relative to the brine drilling fluid. When the salt concentration was greater than 16%, the nanosilica had a negative effect on the drilling fluid, probably because the high salt concentration not only inhibited the hydration of the soil but also caused the nanoparticles to lose their dispersion ability and exhibit aggregation, flocculation and stratification; they could not form a continuous and dense mud cake, leading to an increase in filtration.

#### 5% nanosilica effect on SWM-B properties

**Figure 12:**
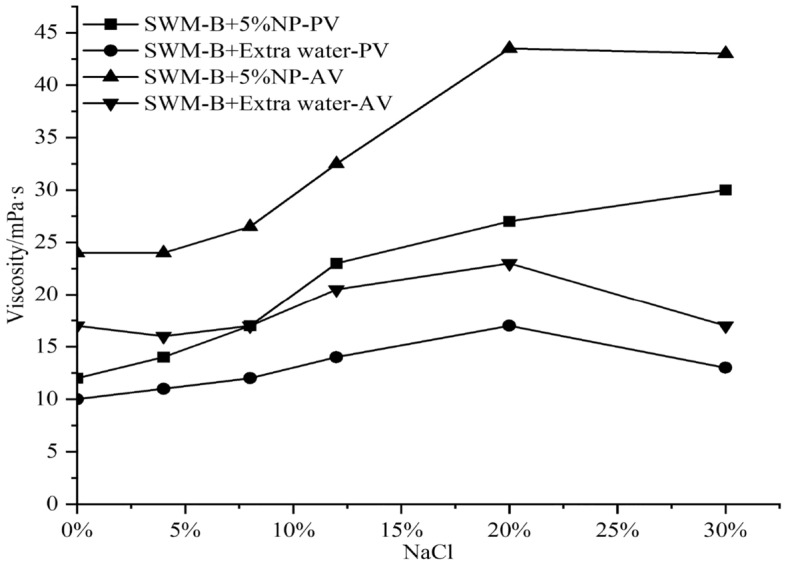
Effect of 5% nanosilica on the viscosity of SWM-B.

**Figure 13:**
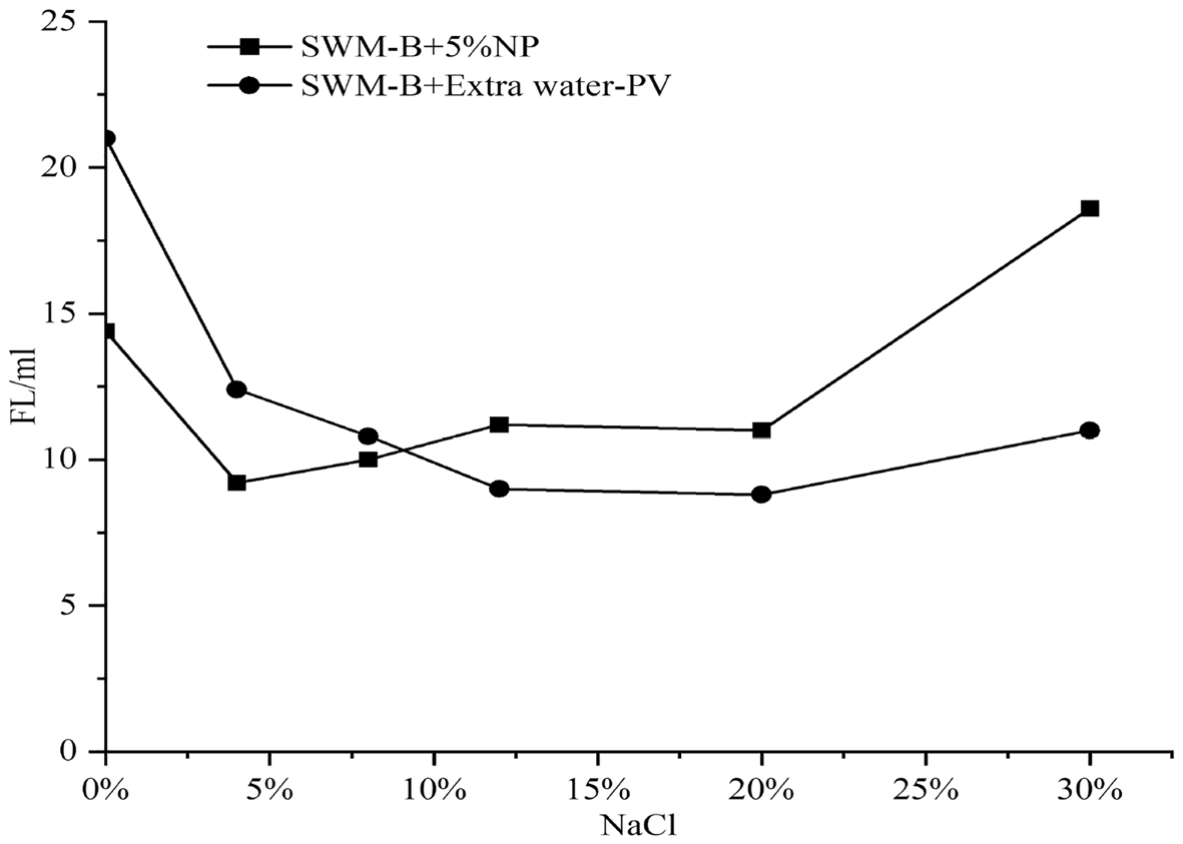
Effect of 5% nanosilica on filtration of SWM-B.

The viscosity and filtration experiments were conducted by adding 5% nanosilica to SWM-B, and the results were obtained as shown in Figs. 12 and 13.

Figures 12 and 13 show that the effect laws of nanosilica on the viscosity of SWM-B were similar. With increasing salt concentration, the viscosity of SWM-B with a 5% concentration of nanosilica increased. However, the viscosity of SWM-B without nanosilica material first increased slowly and then decreased obviously. The effect law of 5% nanosilica on the filtration of SWM-B was similar to that of 3% nanosilica, except when the salt concentration was above 8%, a negative effect appeared, which further indicated that the excessive addition of nanomaterials was not conducive to keeping the performance of brine drilling fluid stable.

#### Influence of nanosilica on the filtration property of SWM-B

**Figure 14:**
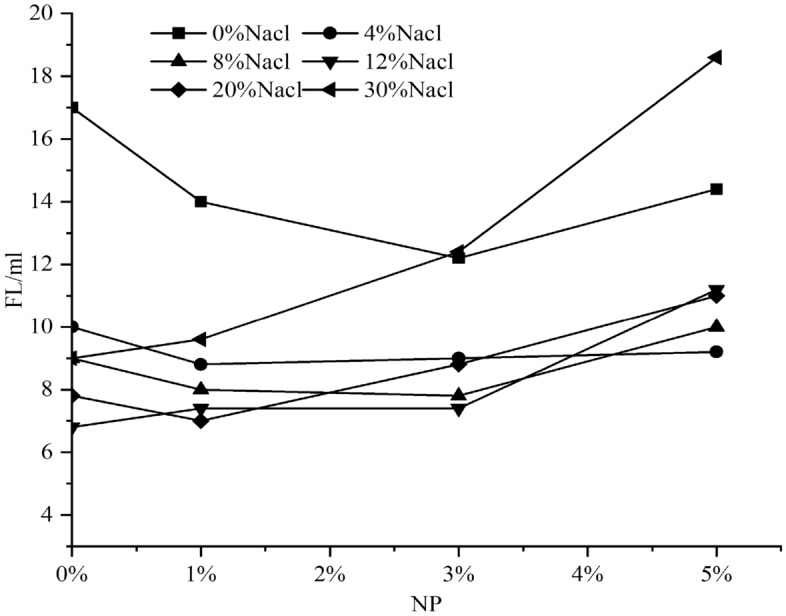
Effect of nanosilica concentration on the filtration of SWM-B.

By integrating the filtration amounts of SWM-B brine drilling fluid under different concentrations of nanosilica, the trend of the variation in filtration properties of brine drilling fluid with the change in nanosilica content was obtained, as shown in Fig. 14.

As shown in Fig. 14, the improvement in the plugging ability of the saltwater drilling fluid SWM-B by nanosilica has a limited range, and when the saltwater resistance is exceeded, negative effects would occur. The plugging capacities of nanoparticles were used to evaluate the stability of nanoparticles in the brine drilling fluid. When the salt concentration was lower than 8%, the larger the concentration of nanosilica and the better the plugging capacity of the brine drilling fluid. However, when the salt concentration was higher than 8%, the higher the concentration of nanosilica and the higher the filtration capacity. This finding indicated that the plugging capacity of saltwater drilling fluid enhanced by nanosilica (1–5%) had an optimal value for NaCl concentration; that is, the NaCl concentration was 0–8%.

#### Effect of nanosilica on the expansion of SWM-B

**Figure 15:**
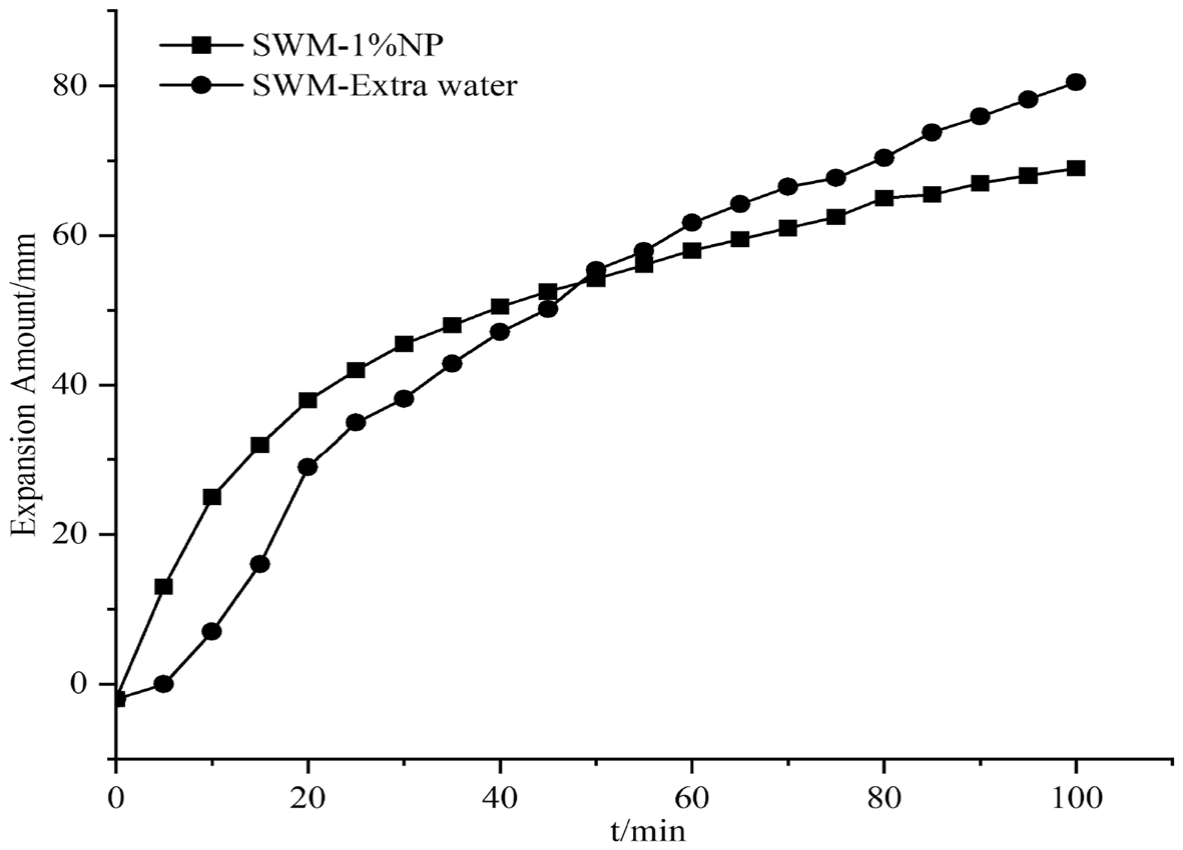
Expansion amount of 1% nanosilica-based slurry and its corresponding water.

**Figure 16:**
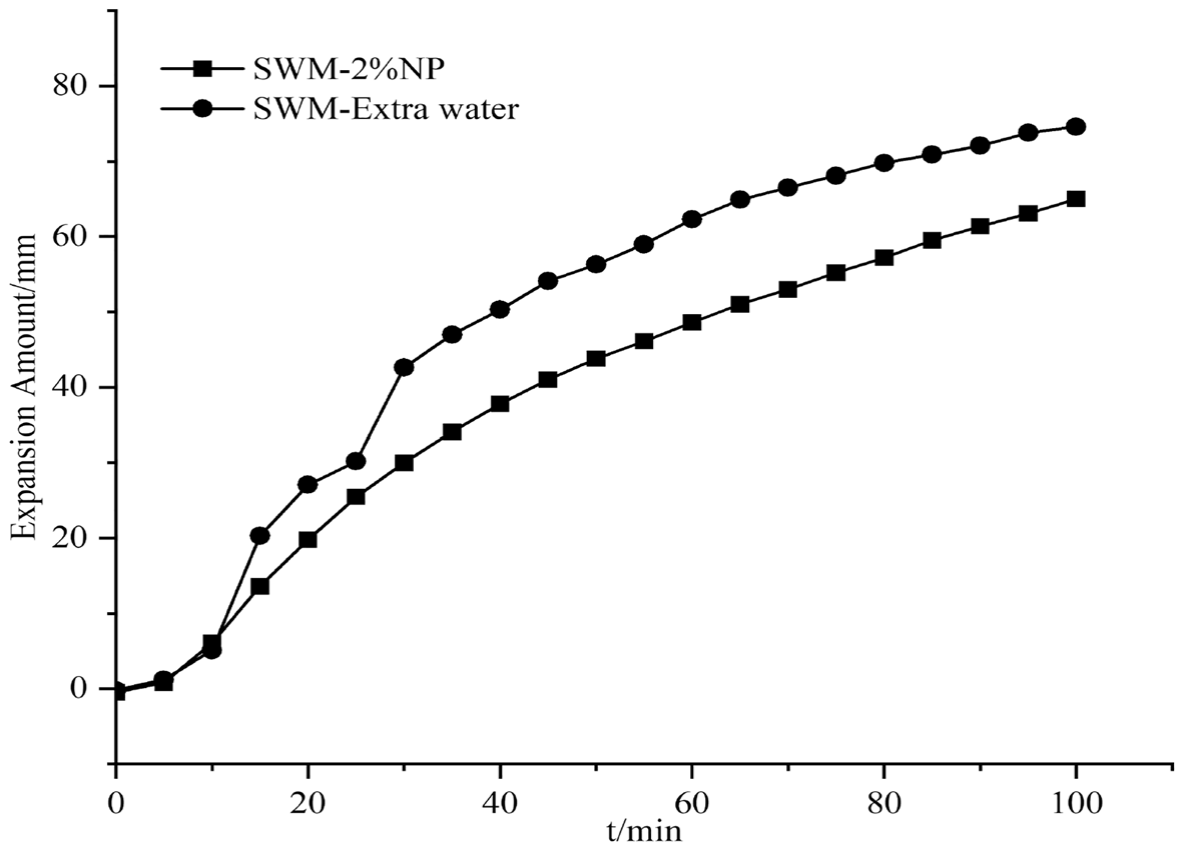
Expansion amount of 2% nanosilica-based slurry and its corresponding water.

**Figure 17:**
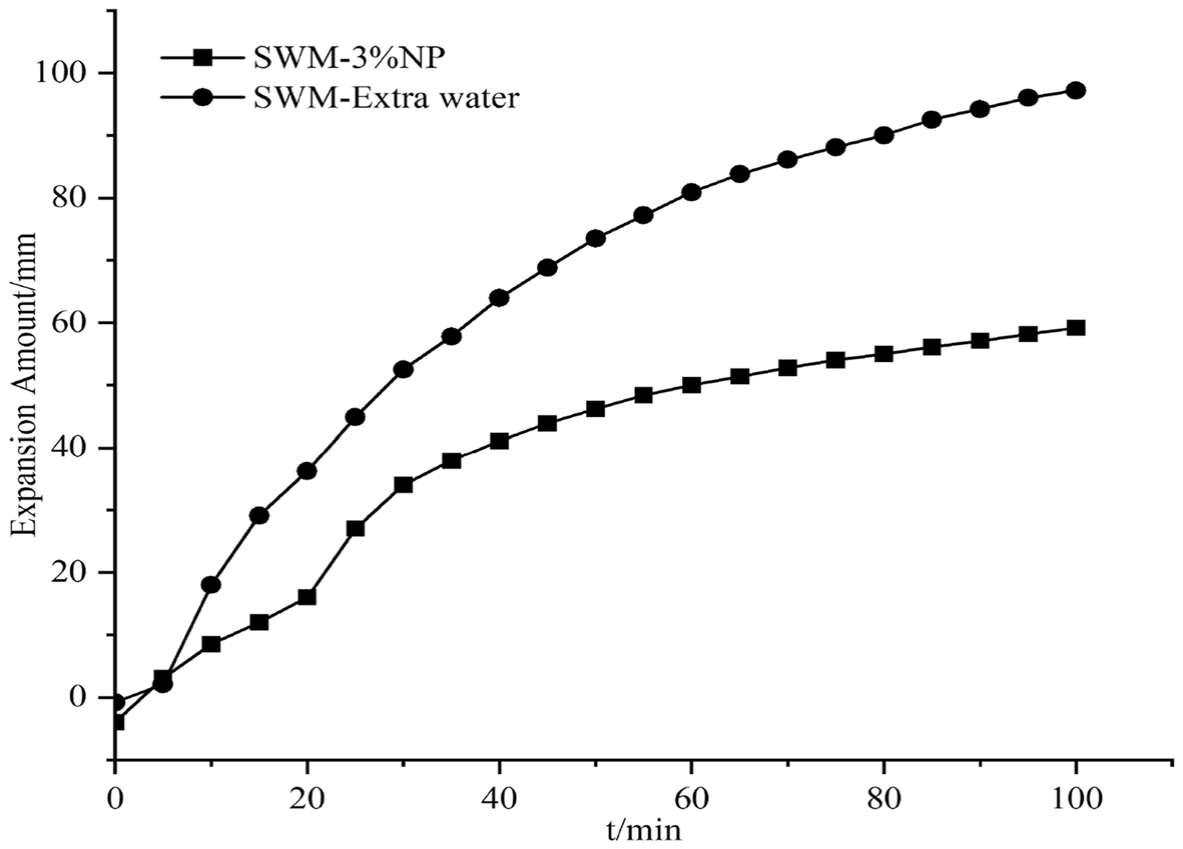
Expansion amount of 3% nanosilica-based slurry and its corresponding water.

**Figure 18:**
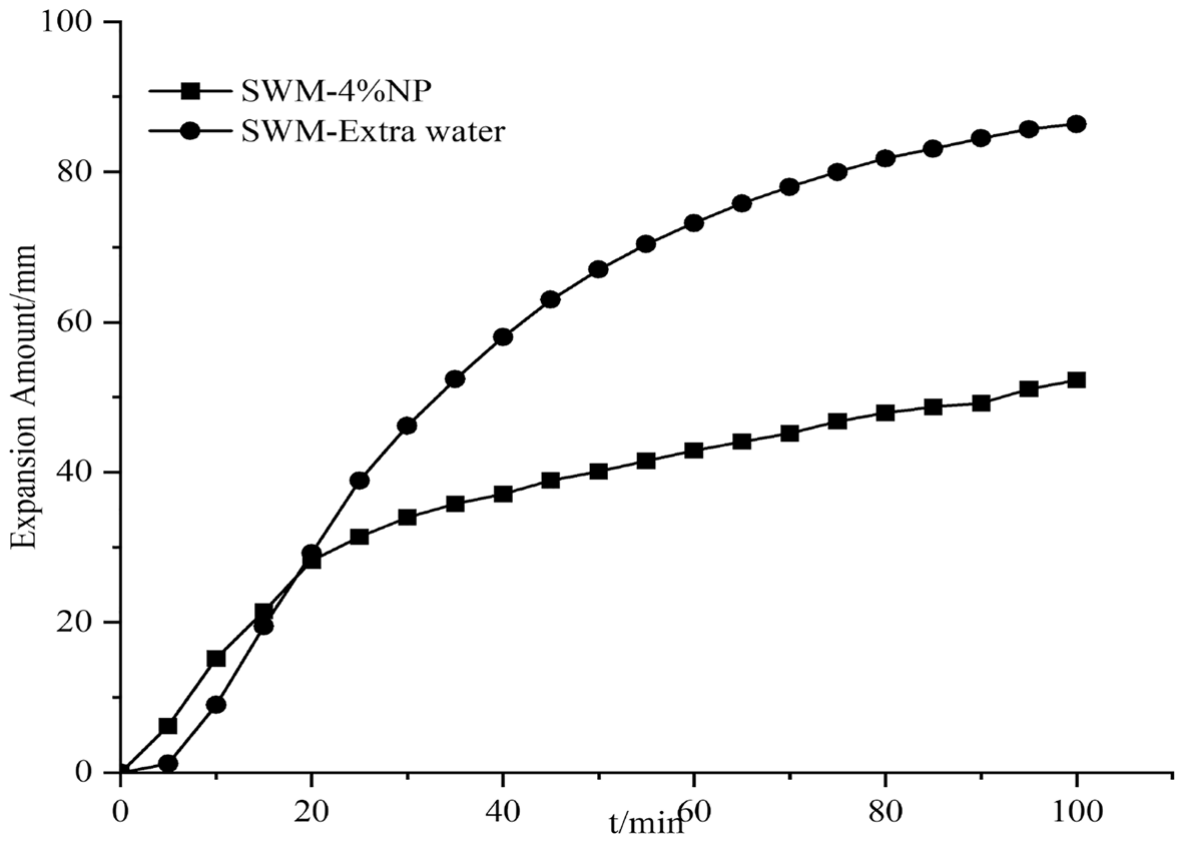
Expansion amount of 4% nanosilica-based slurry and its corresponding water.

**Figure 19:**
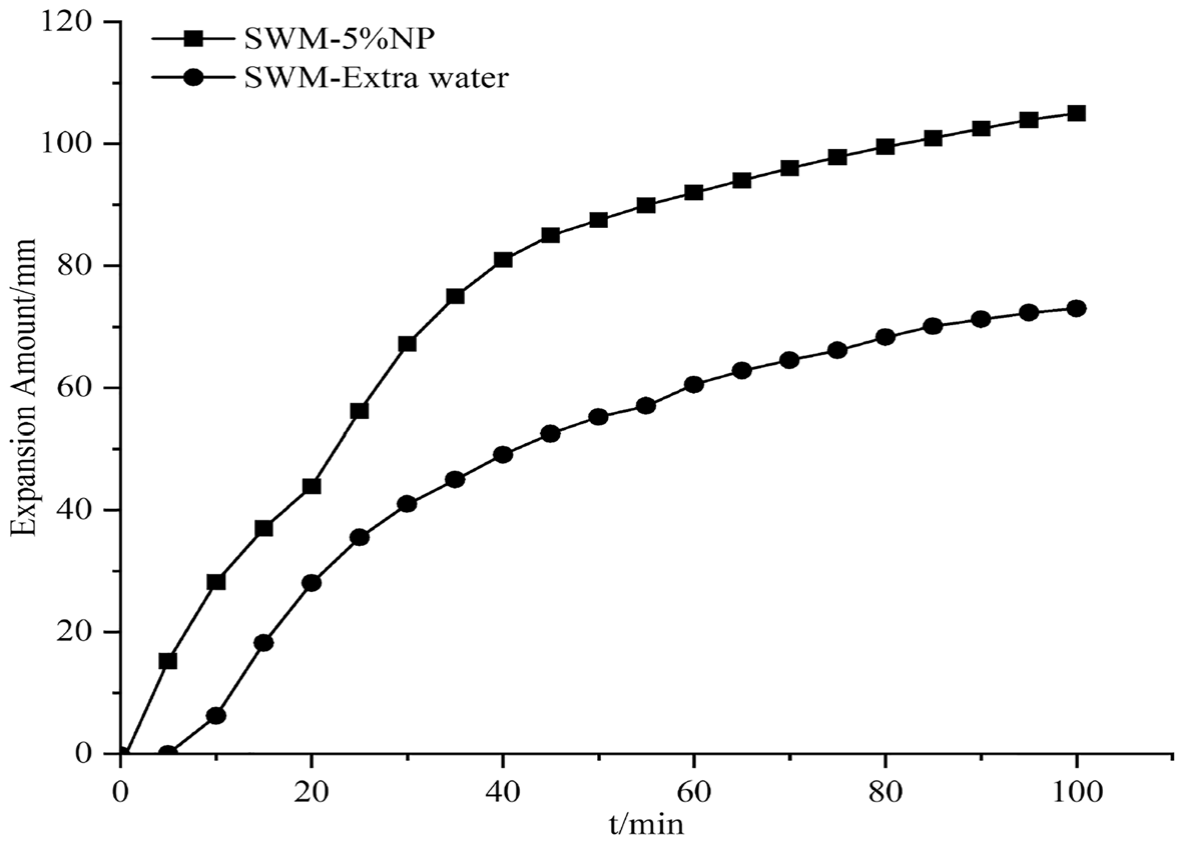
Expansion amount of 5% nanosilica-based slurry and its corresponding water.

**Figure 20:**
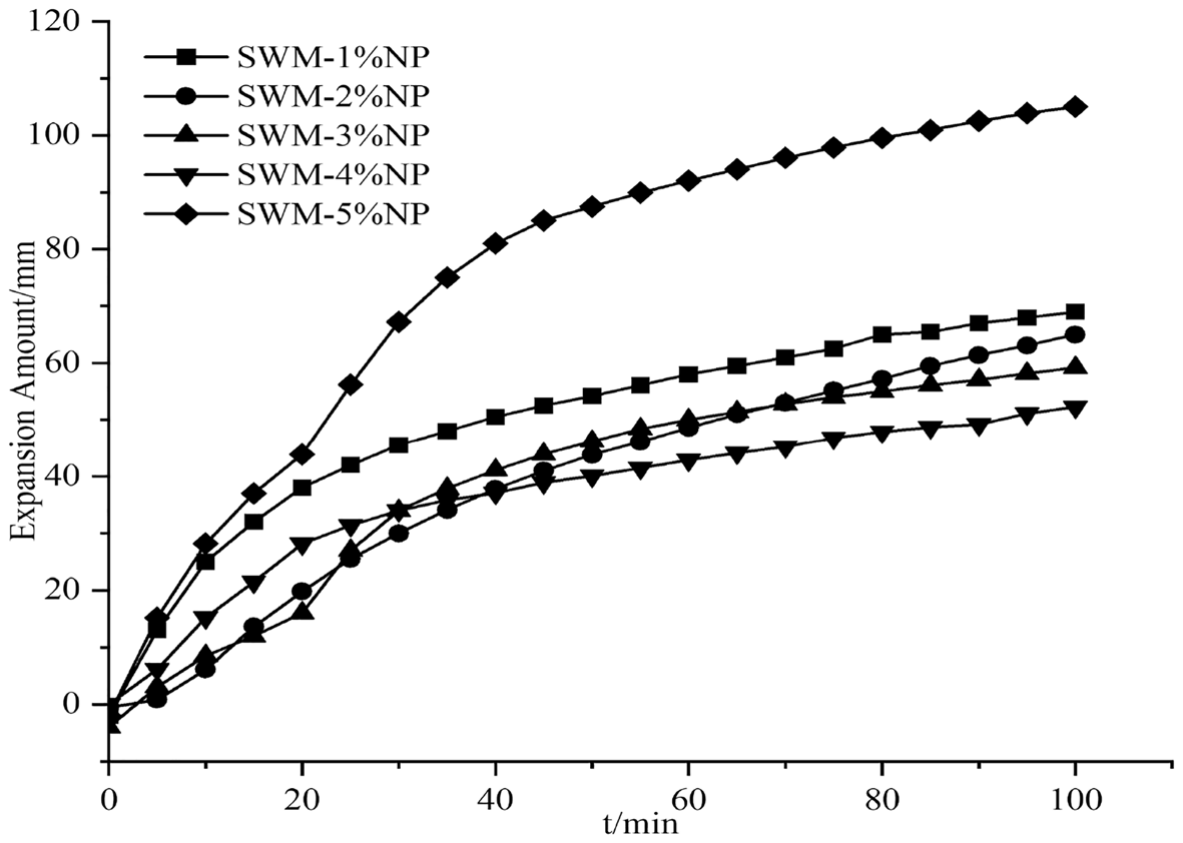
Effect of different concentrations of nanosilica on the expansion amount of brine drilling fluid.

The expansion amount experiments were conducted by adding different concentrations of silica nanomaterials to the salt-resistant drilling fluid, and since the silica dispersion itself contained water, an equal amount of water was added to the drilling fluid; this process was conducted to exclude the effect of water, which was called extra water. The results of the effect of silica nanomaterials on the expansion amount are shown in Figs. 15, 16, 17, 18, 19 and 20.

From Figs. 15, 16, 17, 18, 19 and 20, the addition of nanosilica had a significant effect on inhibiting clay swelling when the nanosilica concentration was below 5%. When the nanosilica content was between 1 and 4%, the increase in nanosilica content inhibited the improvement in performance, but when the content reached 5%, the expansion amount suddenly and rapidly increased. Nanosilica could increase the inhibition performance of brine drilling fluid and reduce the amount of clay expansion, but it should not exceed 5%; otherwise, it would have a negative effect.

## Conclusions

1. Nanosilica has a significant improvement effect on the performance of saline drilling fluid with anti-saline soil configuration and a negative effect on the performance of bentonite brine drilling fluid configuration.
2. In SWM-B brine-based drilling fluid, nanosilica particles can be deposited on the surface of filter cake effectively, the pores of filter paper can be sealed, and filtration loss can be significantly reduced. When the concentration of nanosilica is 1–5%, the optimal concentration of NaCl for its plugging capacity is 0–8%.
3. The addition of nanosilica can effectively improve the inhibition performance of brine-based drilling fluid and reduce the amount of clay swelling. However, the amount of nanosilica does not easily exceed 5%; otherwise, it has a negative impact.
4. 1–5% concentration of nanosilica can effectively improve the viscosity of brine drilling fluid, but the improvement effect is sensitive to the change in salt concentration.
5. Based on the experimental results, the optimal addition amount of silica is 3%, and the salt tolerance reaches 16%. In addition, considering the cost performance of the drilling fluid formula, 3% NP + 4% NaCl + SWM-B is selected as the best formula of brine-based drilling fluid. This formula can be used as a reference for drilling projects in shale or salt formations.

## Data Availability

The datasets generated and analysed during the current study are not publicly available due to the data management policies of the institution, but they are available from the corresponding author upon reasonable request.
